# Profiles of Elderly Patients with Obesity Hypoventilation Syndrome in Martinique: A Single-Center Study

**DOI:** 10.3390/jpm13071089

**Published:** 2023-06-30

**Authors:** Moustapha Agossou, Nadine Simo-Tabué, Marion Dufeal, Bérénice Awanou, Mathilde Provost, Ketty Smith, Elena Badaran, Adel Zouzou, Nelly Ahouansou, Maturin Tabué-Teguo, Moustapha Dramé

**Affiliations:** 1 Department of Respiratory Medicine, CHU of Martinique, 97261 Fort-de-France, France; 2 Department of Geriatrics, CHU of Martinique, 97261 Fort-de-France, France; 3 EpiCliV Research Unit, Faculty of Medicine, University of the French West Indies, 97261 Fort-de-France, France; 4 Department of Clinical Research and Innovation, CHU of Martinique, 97261 Fort-de-France, France

**Keywords:** elderly, obesity hypoventilation syndrome, chronic respiratory failure, chronic hypercapnia, Afro-Caribbean descent

## Abstract

Obesity hypoventilation syndrome (OHS) is a form of chronic respiratory insufficiency related to obesity that affects young and old people. Age appears to be associated with poorer response to treatment by nighttime ventilation. This study aimed to describe the characteristics of elderly subjects (>65 years) with OHS compared to younger patients, with a view to adapting therapy in older individuals. We conducted a retrospective study comparing socio-demographic, clinical, functional characteristics as well as treatment and outcomes between young (<65 years) and older (65 years and older) individuals with OHS at the University Hospital of Martinique. We included 143 patients (114 women), of whom 82 were 65 years or older (57%). Charlson index was higher in the older group. Patients in ≥65 years group were less frequently obese, but more frequently had diabetes mellitus, cardiac arrythmia and arterial hypertension compared to younger patients. There was no difference in the circumstances of diagnosis or arterial blood gas at diagnosis. At follow up, partial pressure of carbon dioxide (pCO_2_) was higher in ≥65 years group. Despite comparable NIV settings, apart from lower expiratory positive airway pressure (EPAP) with higher apnea-hypopnea index (AHI), patients in the ≥65-year-old group remained more frequently hypercapnic. In conclusion, over half (57%) of patients with OHS in our cohort were aged over 65 years. Older patients developed OHS at lower BMI levels than their younger counterparts, and more frequently, had comorbidities such as diabetes, hypertension and cardiac arrhythmia. Increased Charlson index, lower BMI and female sex were independent factors associated with OHS in the elderly.

## 1. Introduction

Obesity is increasingly prevalent around the world. Indeed, the World Health Organization (WHO) estimates that around 4 million deaths per year can be attributed to obesity globally [1]. Obesity hypoventilation syndrome (OHS) is a form of chronic respiratory insufficiency related to obesity. It is defined as the simultaneous presence of obesity (namely, body mass index (BMI) of ≥30 kg/m^2^) and daytime hypercapnia (pCO_2_ > 45 mmHg), in the absence of other causes of alveolar hypoventilation [2]. OHS affects both young and old individuals, and the average age at diagnosis is 60 to 70 years [3,4,5]. OHS is strongly associated with obstructive sleep apnoea, and numerous cardiovascular and metabolic comorbidities [2,6]. It is responsible for substantial healthcare consumption, and carries a high mortality burden [7,8,9]. The management of OHS is based on lifestyle and therapeutic measures aimed primarily at achieving weight loss [10]. In addition, first line therapy is mainly continuous positive airway pressure (CPAP) [11,12,13]. In a previous study, we observed an average age of 67 ± 15 years at the time of diagnosis of OHS, and that older age was associated with the persistence of hypercapnia under non-invasive ventilation (NIV) [14].

In France, the proportion of the population aged 65 years or older increased from 15% in 1995 to 20% in 2019 [15]. In the French overseas Department of Martinique, an island in the Caribbean with an estimated total population of 364,508 inhabitants in 2019 [16], the national statistics institution (INSEE) projects that the proportion of the population aged 60 years and older will reach 40% by 2030, compared to 30% in mainland France [17]. The INSEE further estimated that in 2019, 53% of the population in Martinique was overweight/obese, and a third were obese [18]. There is thus a large pool of subjects with the potential to develop OHS in the coming years.

To the best of our knowledge, no study to date has investigated the prevalence and the characteristics of OHS in elderly subjects, and especially in the population of Martinique. This information would be important to inform public health policy and improve the management of OHS in this population with high rates of obesity and a high proportion of older persons.

The aim of this study was therefore to report the proportion of patients ≥65 years old among those diagnosed with OHS in Martinique, and to compare them to a younger population (<65 years old) with OHS, in terms of circumstances of diagnosis, comorbidities, anthropometric data, lung functional data, adherence to and efficiency of non-invasive ventilation (NIV).

## 2. Methods

### 2.1. Study Population

We performed an observational, single-center, retrospective study in the Department of Respiratory Medicine of the University Hospital (CHU) of Martinique, which is the referral center for patients with respiratory disease for the whole island, and where all patients with chronic respiratory failure, including OHS, are treated.

### 2.2. Eligibility Criteria

Patients were included from 1 January 2019 to 31 December 2022. All patients followed in our department for OHS and hospitalized in the department during the study period were eligible. Data were retrieved from the database by dedicated research staff using a standardized data collection form.

Inclusion criteria were:

- Age 18 years or older;
- Confirmed diagnosis of OHS, i.e., body mass index (BMI) ≥ 30 Kg/m^2^, daytime partial pressure of carbon dioxide (PCO_2_) ≥ 45 mmHg, and absence of COPD, neuromuscular disease or hypothyroidism, severe chest-wall disorders such as kyphoscoliosis.

We excluded patients with a smoking history of more than 10 pack-years in women or 15 pack-years in men, as this may be associated with obstructive syndrome where associated chronic obstructive pulmonary disease (COPD) cannot be ruled out.

### 2.3. Patient Follow-Up

Patients had regular follow-ups every 6 to 12 months in our department to optimize NIV settings and monitor comorbidities. Follow-ups consist in admission in hospitalization unit for 3 to 5 days, to optimize the device settings for the home NIV device and to check the management of comorbidities. The objective is to correct hypercapnia by reaching an optimal balance between the increase in inspiratory assistance and leaks.

### 2.4. Initiation of NIV

BIPAP was initiated during hospitalization. Most patients being followed-up were diagnosed in a context of acute decompensation, and BIPAP was initiated at that time. For patients already receiving CPAP, expiratory positive airway pressure (EPAP) was set at a level equal to CPAP minus 2 cm H_2_O. Inspiratory assistance was then programmed to achieve the optimal balance between efficiency and leaks. For patients who were not receiving CPAP, parameters were set empirically, by adjusting according to residual apnoea-hypopnea index (AHI), asynchrony, leaks and patient complaints.

### 2.5. Comorbidities

Comorbidities were assessed based on the biological work-up and the patient’s ongoing treatment. Cardiovascular comorbidities were diagnosed based on electrophysiological and/or echocardiographic findings, and a cardiologist’s opinion. Asthma diagnosis was made with clinical features, obstructive syndrome on spirometry without smoking or professional exposure to rule out COPD. Patients all underwent polygraphy or polysomnography, either before diagnosis and were already receiving CPAP for severe obstructive sleep apnoea, or at the time of diagnosis. Severe obstructive sleep apnoea was defined as an AHI > 30/h.

The outcomes of this study were:

- to report the proportion of patients ≥65 years among the OHS population in Martinique;
- to compare this population to those <65 years old with OHS, in terms of circumstances of diagnosis, comorbidities, anthropometric data, lung functional data, adherence to and efficiency of non-invasive ventilation (NIV).

### 2.6. Data Recorded

We recorded sociodemographic data (age, age at diagnosis, body mass index (BMI)), comorbidities at diagnosis, comorbidities at inclusion in the cohort, treatment initiated (CPAP, NIV, NIV combined with oxygen therapy) and follow-up data on persistent hypercapnia for all patients.

Persistent hypercapnia was defined as a diurnal PaCO_2_ > 45 mmHg (6 KPa) in room air without acute on chronic respiratory failure. We considered the value of arterial gas, as well as treatment data (ventilator settings, interface, leaks, and daily use) during the last routine visit without exacerbation.

### 2.7. Statistical Analysis

Quantitative variables are described as mean ± standard deviation (SD), and were compared using the Student *t* test. Categorical variables are described as number and percentage and were compared using the chi square or Fisher’s exact test. Covariates were compared between patients aged ≥65 years and those aged <65 years. Multivariable analysis was performed with a binary logistic regression, and included all variables with a *p*-value less or equal to 0.10 by bivariable analyses. Goodness-of-fit of the model was assessed using the Hosmer and Lemeshow’s test. Results were presented as odds ratio (OR) and their respective 95% confidence interval (95% CI). Statistical analyses were performed using SAS version 9.4 (SAS Institute Inc., Cary, NC, USA). Tests were considered significant for *p*-values < 0.05.

### 2.8. Ethical Considerations

The study was performed in accordance with the Declaration of Helsinki and was approved by the Institutional Review Board of the University Hospitals of Martinique under the number 2022/158. At each follow-up consultation or hospitalization, patients received an information leaflet explaining that their routine medical data could be used for research purposes, and indicating how they could explicitly oppose this use, if they so desired, in accordance with French legislation governing retrospective studies using routine medical data. No patient explicitly opposed the use of their medical data for the purposes of the present study.

## 3. Results

We included 143 patients with OHS; 114 were women (80%). Among them, 82 patients (57%) were ≥65 years. Patient characteristics and comorbidities are detailed in Table 1. A total of 136 patients were receiving NIV, of whom 80 (59%) were aged 65 or older, 7 patients were receiving CPAP, of whom 2 were 65 years or older.

The duration of follow-up was 5.6 ± 4.1 years overall (5.1 ± 4.3 vs. 4.2 ± 3.8 years in young vs. older populations, respectively, *p* = 0.17). Patients in the >65-year-old group were less frequently obese, and more frequently had comorbidities such as diabetes mellitus, cardiac arrhythmia and hypertension. They also had a greater expiratory reserve volume than their younger counterparts. At the time of diagnosis, there was no difference between groups in the circumstances of diagnosis, or in blood gas parameters. In terms of outcome, patients aged >65 years remained more frequently hypercapnic. Table 2 describes the device settings and efficacy of NIV in the patient population.

By multivariable analysis, three independent variables were significantly associated with age group. Charlson index was higher in older group (OR = 3.43; 95% CI = 2.11–5.58; *p* < 0.0001). BMI was higher in the younger group (OR = 1.11; 95% CI = 1.02–1.20; *p* = 0.02). The older group tended to be more female (OR = 7.75; 95% CI = 1.62–37.03; *p* = 0.01).

The multivariable model had a good calibration and a good discrimination. The R^2^ was 0.65, the c-statistic was 0.92, and the Hosmer and Lemeshow test was no significant (*p* = 0.18)

## 4. Discussion

Patients aged 65 years and older accounted for 57% of the population of patients with OHS followed up at a large reference center in Martinique. In bivariable analysis, they were more often female and had more comorbidities than younger patients, notably diabetes, cardiac arrhythmia and hypertension, with a correspondingly higher Charlson comorbidity index. Obesity was less severe in aged patients, and they had difficulties achieving adequate ventilation—indeed, the older OHS patients more frequently had persistent hypercapnia despite better adherence, and comparable pressure and leaks compared to those aged <65 years. In multivariable analysis, increase Charlson index, lower BMI and female sex was independent factor associated to older age in OHS. In epidemiological terms, OHS affects more women than men in Martinique [14,19], and this female predominance has previously been reported elsewhere [20,21]. Diabetes mellitus, cardiac conditions and hypertension increase with age [22,23,24].

During aging, the respiratory system undergoes anatomical, physiological and immunological changes [25]. In particular, there is a reduction in gaseous exchange, and an increase in interalveolar space, reduced elastic force in the lung and increased residual volume [26]. There is also a drop in pulmonary compliance and an increase in work of breathing, with a reduction in lung volume and weakened respiratory muscles [25,27,28]. These phenomena culminate in a mismatch between ventilation and perfusion [26]. Furthermore, the sensitivity of chemoreceptors declines, thereby altering the adaptive response [29,30].

Abdominal and thoracic obesity leads to a reduction in lung volume and residual functional capacity [31,32]. Abdominal fat is thought to be responsible for an alteration of the mechanics of ventilation, with reduced mobility of the diaphragm, reduced lung compliance, and increased resistance in the lower airways [2]. These different modifications lead to ventilation–perfusion mismatch [33].

Female hormones may also play a role in compounding these difficulties. Indeed, OHS appears to predominantly affect women, especially in certain geographical regions [20,34], as is the case in the present study. The mechanisms behind this finding remain to be elucidated, but may include the potential role of the discontinuation of progesterone production after menopause [35,36].

Together, these phenomena may explain why older persons may develop OHS at lower BMI levels than their younger counterparts. Our results are in accordance with those of Cantero et al., who observed that NIV was efficacious in subjects aged >75 years in terms of expiratory positive airway pressure (EPAP) and AHI [37]. The slightly lower EPAP and higher AHI in the older subjects in our study, compared to younger patients, could be explained by a certain tolerance when setting the parameters on the machines of older individuals. Enhanced knowledge of the features of OHS in older individuals is of paramount importance to enable early implementation of pluriprofessional prevention programs, to limit the pathological consequences of OHS. Age-related anatomical, physiological and immunological changes should be taken into consideration in the diagnostic and therapeutic work-up of OHS.

### Study Strength and Limitations

Our study is original given the paucity of data in the literature describing OHS in older subjects, especially in Martinique. Our findings may help understand the specificities of this population. In addition, our study is population-based, since our center is the only reference center and therefore, has exhaustive recruitment of all patients on the island. Conversely, our study has some limitations, including the retrospective nature, and the descriptive analysis, which did not account for potential confounding factors. A second limitation is the fact that the majority of the population is of Afro-Caribbean descent. This may limit the generalizability to populations of different descent. Indeed, OSA is reportedly more severe, and causes more comorbidities in individuals of African-American origin [38,39,40]. Also, the features of OHS have been reported to vary according to ethnic origin [6].

## 5. Conclusions

OHS is a form of chronic respiratory insufficiency whose prevalence is likely to increase given the growing number of obese people, and population aging. In our cohort, 57% of patients with OHS were aged 65 years and older. Management of OHS in elderly population is complicated by co-morbidities such as diabetes, cardiac arrhythmia and hypertension and when BMI was less severe. These findings are important to identify the features of OHS and potential healthcare needs in older populations. 

## Figures and Tables

**Table 1:** Patient characteristics at OHS diagnosis (bivariable analyses).

Characteristics	N	<65 Years (*n* = 61)	≥65 Years (*n* = 82)	*p* Value
Charlson Comorbidities Index (mean ± sd)		3.3 ± 1.5	6.2 ± 1.4	<0.001
Body mass index (kg/m^2^) (mean ± sd)		45.2 ± 8.7	38.7 ± 6.7	<0.001
Female sex (%)	114	44 (39)	70 (61)	0.06
Type III obesity (%)	69	39 (64)	30 (37)	0.004
Comorbidities				
Cardiac arrhythmia (%)	28	6 (10)	22 (27)	0.017
Pulmonary hypertension (%)	17	6 (10)	11 (13)	0.6
Chronic heart failure (%)	35	11 (19)	24 (42)	0.16
Arterial hypertension (%)	120	45 (74)	75 (91)	0.006
Peripheral arterial disease (%)	12	2 (3)	10 (12)	0.07
Diabetes mellitus (%)	84	29 (48)	55 (67)	0.03
Asthma (%)	35	12 (20)	23 (28)	0.32
Dyslipidemia	40	15 (25)	25 (30)	0.46
Severe OSA	106	49 (80)	57 (70)	0.18
Circumstances of OHS diagnosis				
Acute respiratory failure (%)	109	42 (56)	67 (93)	0.11
Follow-up of sleep apnoea (%)	26	14 (23)	12 (15)	0.27
Arterial blood gas at diagnosis (mean ± sd)				
pH		7.35 ± 0.06	7.34 ± 0.06	0.86
pCO_2_ (mmHg)		56 ± 11	59 ± 11	0.14
pO_2_ (mmHg)		65 ± 12	66 ± 14	0.88
Bicarbonates (mmol/L)		30 ± 5	31 ± 5	0.39
Arterial blood gas at inclusion (mean ± sd)				
pH		7.39 ± 0.03	7.39 ± 0.04	0.90
pCO_2_		44 ± 5	48 ± 6	<0.001
pO_2_		65 ± 12	66 ± 14	0.88
Pulmonary function tests at inclusion (mean ± sd)				
FEV1^γ^ (% of theoretic value)		57 ± 19	58 ± 17	0.86
FEV1/FVC		79 ± 11	78 ± 16	0.4
FVC (% of theoretical value)		60 ± 18	61 ± 18	0.93
TLC (% of theoretical value)		71 ± 15	73 ± 15	0.66
RV (% of theoretical value)		98 ± 30	94 ± 29	0.56
ERV (% of theoretical value)		51 ± 31	85 ± 55	<0.001

CPAP, Continuous positive airways pressure; PaCO_2_, partial pressure of carbon dioxide; PaO_2_, partial pression of oxygen; FEV1, Forced expiratory volume in 1 s; FVC, Forced vital capacity; TLC, Total lung capacity; RV, Residual volume; ERV, Expiratory reserve volume, sd: standard deviation.

**Table 2:** Settings, quality and efficiency of NIV in OHS patients.

Characteristics	<65 Years *n* = 61	≥65 Years *n* = 82	*p*
EPAP (cmH_2_O) (mean ± sd)	9.1 ± 2.1	8.2 ± 1.9	0.04
IPAP (cmH_2_O) (mean ± sd)	20.9 ± 3.3	20.5 ± 3.1	0.59
Support pressure (cmH_2_O) (mean ± sd)	12 ± 2.7	12.2 ± 2.5	0.74
Daily adherence (Hours) (mean ± sd)	5.6 ± 3.2	6.1 ± 3.3	0.41
Adherence < 4 h (%)	11 (28)	15 (31)	1
Median unintentional leaks (L/min) (mean ± sd)	14.1 ± 21.2	13.6 ± 21.8	0.92
AHI (mean ± sd)	1.6 ± 2.4	3.2 ± 5.8	0.04
Hypercapnia (>45 mmHg) (%)	18 (30)	55 (75)	<0.001

AHI, Apnea hypopnea index, EPAP: Expiratory positive airway pressure, IPAP: Inspiratory positive airway pressure, sd: standard deviation. The total number of patients includes seven patients receiving continuous positive airway pressure.

## Data Availability

The datasets generated and/or analyzed during the current study are available from the corresponding author on reasonable request.

## Abbreviations

BMI	Body mass index
CHU	University hospital center
CPAP	continuous positive airways pressure
COPD	chronic obstructive pulmonary disease
EPAP	Expiratory positive airways pressure
ERV	Expiratory reserve volume
FEV1	Forced expiratory volume in 1 s
FVC	Forced vital capacity
IPAP	Inspiratory positive airways pressure
INSEE	Institut National des Statistiques et des Etudes Economiques
mmHg	millimeter of mercury
NIV	non-invasive ventilation
PaCO_2_	partial pression of carbon dioxide
PaO_2_	partial pression of oxygen
OHS	obesity-hypoventilation syndrome
OSA	obstructive sleep apnea
RV	Residual volume
TLC	Total lung capacity

## References

[1] Obesity [Internet]. https://www.who.int/health-topics/obesity.

[2] Masa J.F., Pépin J.-L., Borel J.-C., Mokhlesi B., Murphy P.B., Sánchez-Quiroga M.Á. (2019). Obesity hypoventilation syndrome. Eur. Respir. Rev..

[3] Bouloukaki I., Mermigkis C., Michelakis S., Moniaki V., Mauroudi E., Tzanakis N., Schiza S.E. (2018). The Association Between Adherence to Positive Airway Pressure Therapy and Long-Term Outcomes in Patients with Obesity Hypoventilation Syndrome: A Prospective Observational Study. J. Clin. Sleep Med..

[4] Masa J.F., Benítez I., Sánchez-Quiroga M.Á., de Terreros F.J.G., Corral J., Romero A., Caballero-Eraso C., Alonso-Álvarez M.L., Ordax-Carbajo E., Gomez-Garcia T. (2020). Long-term Noninvasive Ventilation in Obesity Hypoventilation Syndrome Without Severe OSA: The Pickwick Randomized Controlled Trial. Chest.

[5] Cantero C., Adler D., Pasquina P., Uldry C., Egger B., Prella M., Younossian A.B., Soccal P.M., Pépin J.-L., Janssens J.-P. (2020). Long-Term Noninvasive Ventilation in the Geneva Lake Area: Indications, Prevalence, and Modalities. Chest.

[6] Balachandran J.S., Masa J.F., Mokhlesi B. (2014). Obesity Hypoventilation Syndrome: Epidemiology and Diagnosis. Sleep Med. Clin..

[7] Berg G., Delaive K., Manfreda J., Walld R., Kryger M.H. (2001). The use of health-care resources in obesity-hypoventilation syndrome. Chest.

[8] Wilson M.W., Labaki W.W., Choi P.J. (2021). Mortality and Healthcare Use of Patients with Compensated Hypercapnia. Ann. ATS.

[9] Budweiser S., Riedl S.G., Jörres R.A., Heinemann F., Pfeifer M. (2007). Mortality and prognostic factors in patients with obesity-hypoventilation syndrome undergoing noninvasive ventilation. J. Intern. Med..

[10] Kakazu M.T., Soghier I., Afshar M., Brozek J.L., Wilson K.C., Masa J.F., Mokhlesi B. (2020). Weight Loss Interventions as Treatment of Obesity Hypoventilation Syndrome. A Systematic Review. Ann. Am. Thorac. Soc..

[11] Afshar M., Brozek J.L., Soghier I., Kakazu M.T., Wilson K.C., Masa J.F., Mokhlesi B. (2020). The Role of Positive Airway Pressure Therapy in Adults with Obesity Hypoventilation Syndrome. A Systematic Review and Meta-Analysis. Ann. Am. Thorac. Soc..

[12] Soghier I., Brożek J.L., Afshar M., Kakazu M.T., Wilson K.C., Masa J.F., Mokhlesi B. (2019). Noninvasive Ventilation versus CPAP as Initial Treatment of Obesity Hypoventilation Syndrome. Ann. Am. Thorac. Soc..

[13] Mokhlesi B., Masa J.F., Brozek J.L., Gurubhagavatula I., Murphy P.B., Piper A.J., Tulaimat A., Afshar M., Balachandran J.S., Dweik R.A. (2019). Evaluation and Management of Obesity Hypoventilation Syndrome. An Official American Thoracic Society Clinical Practice Guideline. Am. J. Respir. Crit. Care Med..

[14] Agossou M., Barzu R., Awanou B., Bellegarde-Joachim J., Arnal J.-M., Dramé M. (2023). Factors Associated with the Efficiency of Home Non-Invasive Ventilation in Patients with Obesity-Hypoventilation Syndrome in Martinique. J. Clin. Med..

[15] Démographie—France, Portrait Social | Insee [Internet]. https://www.insee.fr/fr/statistiques/4238369?sommaire=4238781.

[16] Recensement De La Population En Martinique: 364,508 Habitants Au 1er Janvier 2019—Insee Flash Martinique—158 [Internet]. https://www.insee.fr/fr/statistiques/6012596.

[17] Seniors En Martinique: Un Enjeu Économique—Insee Analyses Martinique—10 [Internet]. https://www.insee.fr/fr/statistiques/2128984#titre-bloc-2.

[18] Un Tiers Des Martiniquais Sont Limités Dans Leurs Activités Pour Raison De Santé En 2019—Insee Analyses Martinique—46 [Internet]. https://www.insee.fr/fr/statistiques/5390896#titre-bloc-6.

[19] Agossou M., Awanou B., Inamo J., Dufeal M., Arnal J.-M., Dramé M. (2023). Association between Previous CPAP and Comorbidities at Diagnosis of Obesity-Hypoventilation Syndrome Associated with Obstructive Sleep Apnea: A Comparative Retrospective Observational Study. J. Clin. Med..

[20] BaHammam A.S., Pandi-Perumal S.R., Piper A., Bahammam S.A., Almeneessier A.S., Olaish A.H., Javaheri S. (2016). Gender differences in patients with obesity hypoventilation syndrome. J. Sleep Res..

[21] Castillejo E.O., de Lucas Ramos P., Martin S.L., Barrios P.R., Rodríguez P.R., Caicedo L.M., Cano J.M.B., Gonzalez-Moro J.M.R. (2015). Noninvasive mechanical ventilation in patients with obesity hypoventilation syndrome. Long-term outcome and prognostic factors. Arch. Bronconeumol..

[22] Nakano T., Ito H. (2007). Epidemiology of diabetes mellitus in old age in Japan. Diabetes Res. Clin. Pract..

[23] Duncan A.K., Vittone J., Fleming K.C., Smith H.C. (1996). Cardiovascular disease in elderly patients. Mayo Clin. Proc..

[24] Benetos A., Petrovic M., Strandberg T. (2019). Hypertension Management in Older and Frail Older Patients. Circ. Res..

[25] Sharma G., Goodwin J. (2006). Effect of aging on respiratory system physiology and immunology. Clin. Interv. Aging.

[26] Janssens J.P., Pache J.C., Nicod L.P. (1999). Physiological changes in respiratory function associated with ageing. Eur. Respir. J..

[27] Lowery E.M., Brubaker A.L., Kuhlmann E., Kovacs E.J. (2013). The aging lung. Clin. Interv. Aging.

[28] Zaugg M., Lucchinetti E. (2000). Respiratory function in the elderly. Anesthesiol. Clin. N. Am..

[29] Johnson B.D., Reddan W.G., Seow K.C., Dempsey J.A. (1991). Mechanical constraints on exercise hyperpnea in a fit aging population. Am. Rev. Respir. Dis..

[30] Johnson B.D., Reddan W.G., Pegelow D.F., Seow K.C., Dempsey J.A. (1991). Flow limitation and regulation of functional residual capacity during exercise in a physically active aging population. Am. Rev. Respir. Dis..

[31] Hodgson L.E., Murphy P.B., Hart N. (2015). Respiratory management of the obese patient undergoing surgery. J. Thorac. Dis..

[32] Pépin J.L., Timsit J.F., Tamisier R., Borel J.C., Lévy P., Jaber S. (2016). Prevention and care of respiratory failure in obese patients. Lancet Respir. Med..

[33] Zavorsky G.S., Hoffman S.L. (2008). Pulmonary gas exchange in the morbidly obese. Obes. Rev..

[34] Barbagelata E., Ambrosino I., de Terán T.D., González M., Nicolini A., Banfi P., Ferraioli G., Solidoro P. (2022). Gender differences in obesity hypoventilation syndrome: A concise narrative review. Minerva Med..

[35] BaHammam A.S., Almeneessier A.S. (2019). Is Obesity Hypoventilation Syndrome A Postmenopausal Disorder?. Open Respir. Med. J..

[36] Anttalainen U., Saaresranta T., Vahlberg T., Polo O. (2014). Short-term medroxyprogesterone acetate in postmenopausal women with sleep-disordered breathing: A placebo-controlled, randomized, double-blind, parallel-group study. Menopause.

[37] Cantero C., Adler D., Pasquina P., Uldry C., Egger B., Prella M., Younossian A.B., Soccal-Gasche P., Pépin J.-L., Janssens J.-P. (2020). Long-Term Non-invasive Ventilation: Do Patients Aged over 75 Years Differ from Younger Adults?. Front. Med..

[38] Thornton J.D., Dudley K.A., Saeed G.J., Schuster S.T., Schell A., Spilsbury J.C., Patel S.R. (2022). Differences in Symptoms and Severity of Obstructive Sleep Apnea between Black and White Patients. Ann. Am. Thorac. Soc..

[39] Andreozzi F., Van Overstraeten C., Ben Youssef S., Bold I., Carlier S., Gruwez A., André S., Bruyneel A.-V., Bruyneel M. (2020). African ethnicity is associated with a higher prevalence of diabetes in obstructive sleep apnea patients: Results of a retrospective analysis. Sleep Breath..

[40] Johnson D.A., Guo N., Rueschman M., Wang R., Wilson J.G., Redline S. (2018). Prevalence and correlates of obstructive sleep apnea among African Americans: The Jackson Heart Sleep Study. Sleep.

